# The combined association of physical activity and alcohol use with long-term mortality: an age-stratified analysis

**DOI:** 10.1186/s12889-024-19326-8

**Published:** 2024-07-08

**Authors:** Bingqi Fu, Yu Yu, Sijing Cheng, Hao Huang, Tianxin Long, Juwei Yang, Chi Cai, Min Gu, Hongxia Niu, Wei Hua

**Affiliations:** Cardiac Arrhythmia Center, National Center for Cardiovascular Diseases, State Key Laboratory of Cardiovascular Disease, Fuwai Hospital, Chinese Academy of Medical Sciences and Peking Union Medical College, No. 167 Bei Li Shi Rd, Xicheng District, Beijing, 100037 China

**Keywords:** Physical activity, Alcohol, Combined association, Mortality, National health and nutrition examination survey

## Abstract

**Background:**

The combined association of physical activity (PA) and alcohol use (AU) with long-term mortality is yet to be investigated.

**Methods:**

For the current study, 12,621 participants aged ≥ 20 years were enrolled from the National Health and Nutrition Examination Survey (1999–2004). The study endpoint was all-cause mortality. Cox proportional hazards regression models were used to examine the combined effect of PA and AU on long-term mortality.

**Results:**

The study population was divided into young (< 60 years, *N* = 8,258) and old (≥ 60 years, *N* = 4,363) groups. The median follow-up time was 203 months. In both young and old group, sedentary lifestyle combined with even minimal AU were associated with elevated risk of death (all *P* < 0.05). In young group, the integration of high volume AU with any degree of PA, including sedentary PA (HR = 2.35, 95% CI 1.24–4.44, *P* = 0.009), low PA (HR = 1.64, 95% CI 1.01–2.68, *P* = 0.047), and moderate-to-vigorous PA (HR = 1.99, 95% CI 1.03–3.84, *P* = 0.041), was associated with an increased risk of mortality. This relationship persisted as significant after adjusting for potential confounders (all *P* < 0.05). In old group, combining moderate-to-vigorous PA and low volume AU (HR = 0.59, 95% CI 0.37–0.94, *P* = 0.027) was associated with a reduction in mortality. After adjustment, the combination of moderate-to-vigorous PA and low volume AU was independently associated with favorable prognostic outcomes (all *P* < 0.05).

**Conclusions:**

In both age groups, combining sedentary lifestyle with even minimal AU was a risk factor for death. In young group, combining any level of PA with high volume AU was associated with increased mortality. In old group, combining moderate-to-vigorous PA with low volume AU was related to reduced mortality.

**Supplementary Information:**

The online version contains supplementary material available at 10.1186/s12889-024-19326-8.

## Background

Modifiable lifestyle factors are closely associated with life expectancy and the incidence of chronic disease [1–3]. Advocating for a combination of healthy lifestyle factors is a fundamental aspect of public health strategies aimed at minimizing early mortality [4]. Physical activity (PA) intensity is correlated with better outcomes. Engaging in regular exercise that meets or surpasses the current PA guidelines is linked to a reduced risk of all-cause mortality [5–8]. Alcohol use (AU), on the other hand, exhibited a J-shaped curve in its association with adverse clinical outcomes [9–11].

The behavior of PA and AU are positively correlated, suggesting that higher levels of PA are often associated with greater AU, and vice versa. Physically active individuals that engage in problematic drinking might appear “healthy”, masking alcohol-related issues and precipitating future personal and societal risks [12]. Previous research has examined the interplay between PA and AU, concentrating primarily on AU’s impact on exercise performance [13–15], and the influence of exercise on alcohol dependency and various mental health disorders [16–19]. However, the combined effect of PA and AU on clinical outcomes is yet unknown. Besides, age-stratified analysis is necessary, given the significantly different mortality risks faced by young and old populations [20, 21]. Therefore, this study was designed to explore the combined association of PA and AU on long-term, all-cause mortality, with a novel stratification by age groups.

## Methods

### Study design and population

The National Health and Nutrition Examination Survey (NHANES) is a program conducted every 2 years and employs a complex, multistage probability sampling design to select participants representative of the civilian, non-institutionalized population of the United States to assess the health and nutritional status of adults and children. Sample weights are assigned to all NHANES participants, accounting for the probability of selection, nonresponse adjustments, and post-stratification adjustments. NHANES is conducted by the Centers for Disease Control and Prevention and the National Center for Health Statistics (NCHS). The NCHS Research Ethics Review Committee reviewed and approved the NHANES study protocol.

**Fig. 1:**
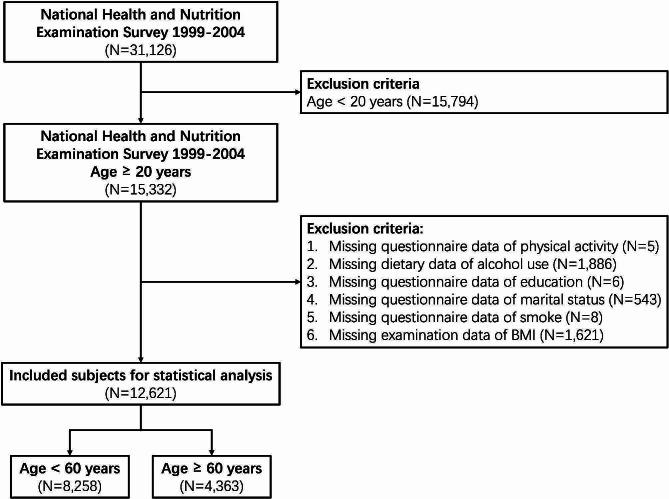
Flowchart of the study population

For the current study, data from three cycles, 1999–2000, 2001–2002, and 2003–2004, were merged (available at: https://wwwn.cdc.gov/nchs/nhanes/Default.aspx). For the 1999–2000 and 2001–2002 cycle, the dietary day one 4-year sample weight was applied. For the 2003–2004 cycle, the dietary day one sample weight was applied. There were 31,126 participants in NHANES 1999–2004. After excluding age < 20 years (*N* = 15,794), missing questionnaire data of PA (*N* = 5), missing dietary data of AU (*N* = 1,886), missing questionnaire data of education (*N* = 6), missing questionnaire data of marital status (*N* = 543), missing questionnaire data of smoking (*N* = 8), and missing examination data of body mass index (BMI, *N* = 1,621), 12,621 participants were included in the final analysis (Fig. 1).

### Data collection and definition

Demographic data were extracted including age, sex, race, education, marital status, and smoking status. In addition, examination and laboratory data were extracted including height, weight, BMI, systolic and diastolic blood pressure, white blood cell count, hemoglobin, lymphocyte count, platelet count, high-density and low-density lipoprotein cholesterol, total cholesterol, serum creatinine, total bilirubin, albumin, and glycohemoglobin. Furthermore, questionnaire data were extracted including PA, and past medical history of congestive heart failure, coronary heart disease, angina, myocardial infarction, stroke, cancer, hypertension, hypercholesterolemia, diabetes mellitus, and chronic kidney disease. Eventually, dietary data were extracted including AU.

The participants’ daily PA levels were classified as sedentary, low, moderate, or vigorous according to the questionnaire data. Participants were assumed to have a sedentary life if they sat during the day and did not walk about very much. Participants were assumed to have low PA if they walked a lot during the day but did not carry or lift things very often. Participants were assumed to have moderate PA if they lifted light loads or climbed stairs or hills often. Participants were assumed to have vigorous PA if they did heavy work or carried heavy loads.

The participants’ AU levels were classified based on the dietary intake of alcohol per day. Occasional drinkers had < 1.3 g AU, low-volume drinkers had 1.3–24.0 g AU, medium-volume drinkers had 25.0–44.0 g AU, and high-volume drinkers had ≥ 45.0 g AU [11].

### Follow-up and study endpoints

The NHANES participants were followed up for mortality until 31 December 2019. The follow-up time was calculated using person months from the date of interview to the date of death or the end of the follow-up period. The median follow-up time was 203 months (or ~ 16.9 years). The endpoint of the current study was all-cause mortality. Death data were extracted from public-use linked mortality files in the NHANES database (available at: https://www.cdc.gov/nchs/data-linkage/mortality-public.htm).

### Statistical analysis

All statistical analyses were conducted in accordance with guidelines from the Centers for Disease Control and Prevention (available at: https://wwwn.cdc.gov/nchs/nhanes/tutorials/default.aspx). Sample weights were assigned to participants as recommended by NCHS. The baseline characteristics of the study population are presented as weighted samples. The continuous variables are presented as weighted mean (standard deviation). The categorial variables are presented as unweighted number (survey-weighted percentages).

The study population was categorized into young (< 60 years) and old (≥ 60 years) groups, and the baseline characteristics of each group were subclassified by PA and AU levels. The continuous variables were compared using the weighted linear regression models and the categorial variables were compared using the weighted chi-square tests. Bar charts were used to show all-cause mortality per 100,000 person-years in various levels of PA and AU combinations. Univariate Cox proportional hazard regression analysis was conducted to explore the potential risk factors for all-cause mortality. Possible confounding factors (age, race, education, marital status, BMI, smoking status, congestive heart failure, myocardial infarction, stroke, cancer, hypertension, hypercholesterolemia, and diabetes mellitus) were incorporated into multivariate Cox proportional hazard regression models to evaluate the independent effect of PA and AU on all-cause mortality, as well as the combined association of PA and AU with all-cause mortality. The selection of confounding factors was based on clinical relevance and the univariate association with outcomes. Statistical data were analyzed using R statistical software version 4.3.1. Two-tailed P values < 0.05 were considered statistically significant.

## Results

### Baseline characteristics for total population

Overall, 12,621 participants were included in this study. The baseline clinical characteristics of the weighted samples are displayed in Table 1. Generally, the mean age of weighted sample was 46.17 (16.96) years, and 48.0% were male. The median BMI was 28.14 (6.36) kg/m^2^. Non-Hispanic white participants accounted for the majority (72.2%) of the population, followed by participants who were non-Hispanic black (10.8%), Mexican American (7.3%), other Hispanic (5.1%), and other races (4.5%). In terms of education and marital status, 53.9% of the participants had attained an education level beyond high school, and 57.5% of the participants reported being married. Never smokers comprised 50.0% of the population, former smokers accounted for 25.6%, and current smokers made up 24.4%. About half of the participants had a low daily PA and occasional AU.

**Table 1:** Baseline characteristics of NHANES participants, 1999–2004

Characteristics	Total (*N* = 12,621)	Age < 60 years (*N* = 8258)	Age ≥ 60 years (*N* = 4363)	*P* value
	**Unweighted N (weighted percentage)**			
**Male gender**	5999 (48.0)	3828 (49.0)	2171 (44.7)	< 0.001
**Race**				< 0.001
Non-Hispanic White	6382 (72.2)	3875 (69.4)	2507 (81.4)	
Non-Hispanic Black	2391 (10.8)	1699 (11.7)	692 (8.0)	
Mexican American	2837 (7.3)	1930 (8.6)	907 (3.3)	
Other Hispanic	568 (5.1)	420 (5.4)	148 (4.0)	
Others	443 (4.5)	334 (4.9)	109 (3.3)	
**BMI group**				< 0.001
Underweight	200 (1.9)	146 (2.1)	54 (1.3)	
Normal	3824 (32.4)	2623 (33.8)	1201 (27.7)	
Overweight	4531 (34.0)	2785 (32.5)	1746 (39.1)	
Obese	4066 (31.6)	2704 (31.6)	1362 (31.9)	
**Education**				< 0.001
< high school	4050 (20.3)	2215 (17.6)	1835 (29.3)	
high school	2991 (25.8)	1977 (25.0)	1014 (28.3)	
> high school	5580 (53.9)	4066 (57.4)	1514 (42.5)	
**Marital status**				< 0.001
Married	7169 (57.5)	4584 (56.0)	2585 (62.3)	
Widowed	1218 (6.4)	121 (1.3)	1097 (23.2)	
Divorced	1111 (9.5)	713 (9.5)	398 (9.4)	
Separated	422 (2.8)	332 (3.3)	90 (2.1)	
Never married	2000 (17.9)	1860 (22.5)	140 (2.8)	
Living with partner	701 (5.9)	648 (7.4)	53 (1.1)	
**Smoking status**				< 0.001
Never smoker	6489 (50.0)	4457 (51.2)	2032 (46.6)	
Former smoker	3383 (25.6)	1580 (20.6)	1803 (42.1)	
Current smoker	2748 (24.4)	2221 (28.2)	527 (11.8)	
**Physical activity**				< 0.001
Sedentary	3131 (24.4)	1854 (23.6)	1277 (27.2)	
Low	6727 (50.8)	4237 (48.8)	2490 (57.3)	
Moderate-to-vigorous	2763 (24.8)	2167 (27.6)	596 (15.5)	
**Alcohol use**				< 0.001
Occasional	9954 (76.9)	6300 (75.2)	3654 (82.5)	
Low volume	1093 (9.3)	707 (9.3)	386 (9.2)	
Medium volume	666 (6.0)	499 (6.6)	167 (4.3)	
High volume	908 (7.8)	752 (8.9)	156 (4.1)	
**Past medical history**				
Congestive heart failure	390 (2.4)	81 (1.1)	309 (6.9)	< 0.001
Coronary heart disease	562 (3.9)	109 (1.6)	453 (11.3)	< 0.001
Angina	475 (3.4)	112 (1.6)	363 (9.3)	< 0.001
Myocardial infarction	568 (3.9)	120 (1.8)	448 (10.7)	< 0.001
Stroke	406 (2.5)	90 (1.2)	316 (6.7)	< 0.001
Cancer	1102 (8.6)	302 (4.7)	800 (21.6)	< 0.001
Hypertension	3964 (28.1)	1606 (20.6)	2358 (52.8)	< 0.001
Hypercholesterolemia	3404 (38.5)	1535 (32.3)	1869 (52.5)	< 0.001
Diabetes mellitus	1225 (7.2)	415 (4.6)	810 (15.8)	< 0.001
Chronic kidney disease	223 (1.9)	93 (1.3)	130 (3.9)	< 0.001
	**Weighted mean (SD)**			
Age (years)	46.17 (16.96)	38.77 (11.15)	70.42 (7.43)	< 0.001
BMI (kg/m^2^)	28.14 (6.36)	28.10 (6.60)	28.25 (5.49)	0.334
SBP (mmHg)	124.38 (19.80)	119.52 (15.85)	140.55 (22.82)	< 0.001
DBP (mmHg)	72.07 (13.13)	72.78 (11.97)	69.69 (16.18)	< 0.001
WBC count (/mm^3^)	7.33 (2.29)	7.39 (2.15)	7.17 (2.68)	0.006
Hemoglobin (g/dL)	14.49 (1.47)	14.56 (1.49)	14.25 (1.36)	< 0.001
Lymphocyte count (/mm^3^)	2.14 (1.14)	2.17 (0.73)	2.05 (1.95)	0.006
Platelet count (/mm^3^)	268.24 (66.70)	271.96 (64.98)	256.14 (70.67)	< 0.001
TC (mmol/L)	5.24 (1.12)	5.18 (1.12)	5.44 (1.08)	< 0.001
LDL-C (mmol/L)	3.12 (0.92)	3.09 (0.91)	3.21 (0.95)	< 0.001
HDL-C (mmol/L)	1.40 (0.42)	1.39 (0.41)	1.43 (0.43)	0.068
Total bilirubin (umol/L)	12.21 (5.36)	12.21 (5.21)	12.18 (5.82)	0.827
Albumin (g/L)	43.39 (3.44)	43.70 (3.49)	42.34 (3.05)	< 0.001
Creatinine (umol/L)	75.90 (35.78)	73.45 (32.05)	83.97 (45.02)	< 0.001
Glycohemoglobin (%)	5.48 (0.90)	5.38 (0.84)	5.81 (1.02)	< 0.001

Abbreviations: NHANES, National Health and Nutrition Examination Survey; BMI, body mass index; SBP, systolic blood pressure; DBP, diastolic blood pressure; SD, standard deviation; PA, physical activity; AU, alcohol use; WBC, white blood cell; TC, total cholesterol; LDL-C, low-density lipoprotein cholesterol; HDL-C, high-density lipoprotein cholesterol

The study population was divided into young (< 60 years, *N* = 8,258) and old (≥ 60 years, *N* = 4,363) groups (Fig. 1). Relative to the younger group, the older group exhibited a higher prevalence of chronic diseases, and reduced engagement in moderate-to-vigorous PA and medium-to-high AU.

The baseline characteristics of young and old group by PA and AU levels were presented in Supplementary Tables 1–4. For different PA levels, both young and old participants with moderate-to-vigorous PA tended to be male, had higher proportion of high volume AU, lower proportion of chronic medical disease, lower BMI and glycohemoglobin (all *P* < 0.05). For different AU levels, both young and old participants with high volume AU tended to be male, had higher proportion of current smoker, never married and divorced. (all *P* < 0.05).

### The association between PA and AU with all-cause mortality for age < 60 years

**Fig. 2:**
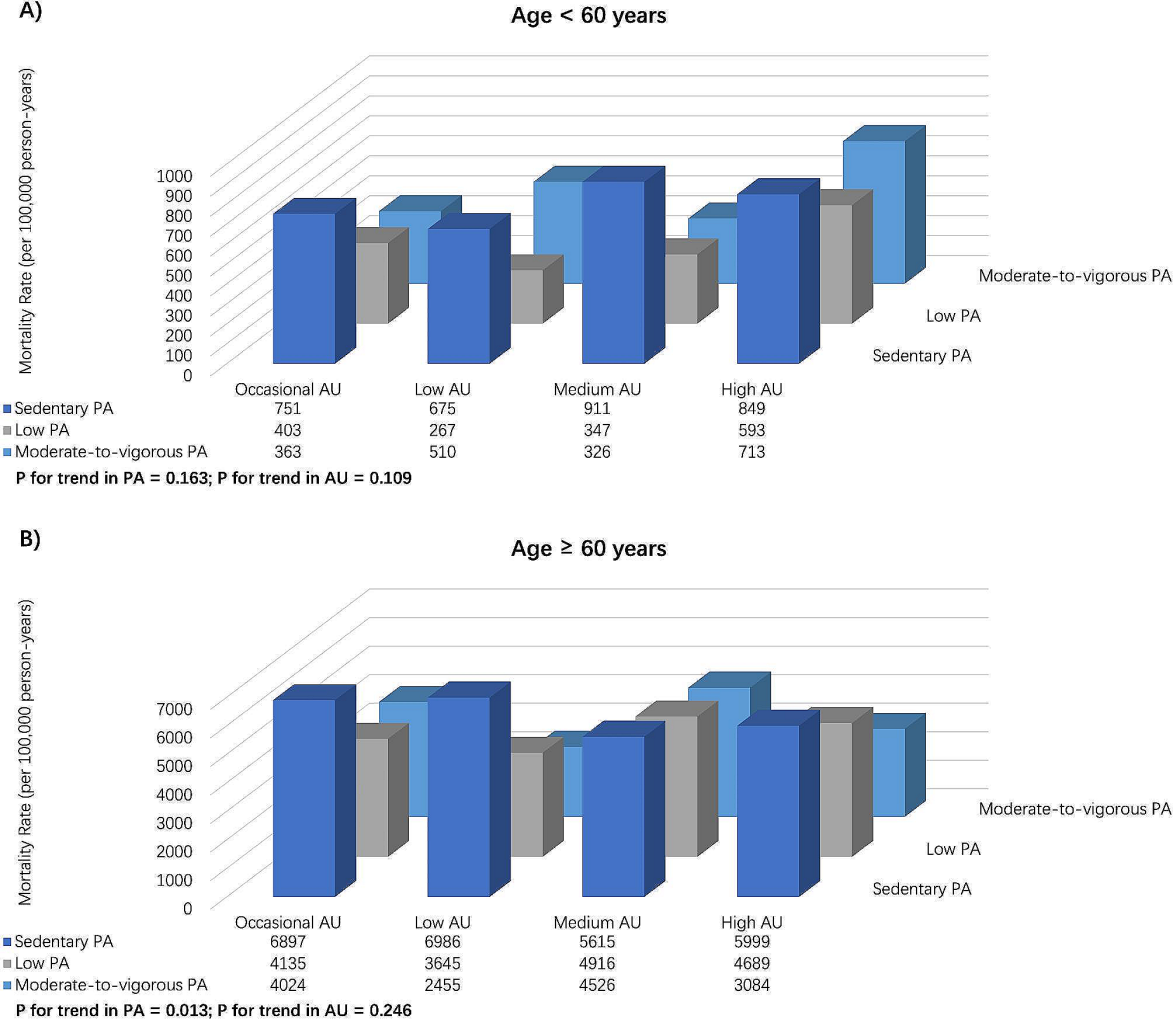
Bar charts for the all-cause mortality rate per 100,000 person-years for various levels of PA and AU combinations. **A**) Age < 60 years; **B**) Age ≥ 60 years. Abbreviations: PA, physical activity; AU, alcohol use

For the younger participants, there were no significant difference for the all-cause mortality per 100,000 person-years among AU levels (P for trend in PA = 0.163, P for trend in AU = 0.109; Fig. 2).

The Cox regression analyses between PA and AU with all-cause mortality for age < 60 years were summarized in Table 2.

**Table 2:** Cox regression analysis of physical activity and alcohol use with all-cause mortality for age < 60 years

Variables	Univariate analysis		Adjusted model 1^a^		Adjusted model 2^b^		Adjusted model 3^c^	
	HR (95% CI)	*P* value	HR (95% CI)	*P* value	HR (95% CI)	*P* value	HR (95% CI)	*P* value
**Physical activity**								
Sedentary	Reference		Reference		Reference		Reference	
Low	0.53 (0.42–0.66)	**< 0.001**	0.53 (0.42–0.66)	**< 0.001**	0.59 (0.44–0.81)	**< 0.001**	0.59 (0.43–0.80)	**< 0.001**
Moderate-to-vigorous	0.54 (0.41–0.72)	**< 0.001**	0.49 (0.36–0.66)	**< 0.001**	0.58 (0.40–0.84)	**0.004**	0.50 (0.35–0.74)	**< 0.001**
**Alcohol use**								
Occasional	Reference		Reference		Reference		Reference	
Low volume	0.92 (0.61–1.38)	0.692	0.94 (0.63–1.40)	0.752	0.87 (0.51–1.49)	0.621	0.85 (0.49–1.48)	0.568
Medium volume	0.97 (0.64–1.45)	0.867	0.89 (0.60–1.32)	0.560	0.67 (0.41–1.10)	0.111	0.63 (0.39–1.02)	0.062
High volume	1.45 (1.10–1.93)	**0.010**	1.10 (0.81–1.49)	0.549	1.89 (1.39–2.57)	**< 0.001**	1.51 (1.10–2.09)	**0.012**
**Combined variables**								
Moderate-to-vigorous PA, occasional AU	Reference		Reference		Reference		Reference	
Moderate-to-vigorous PA, low AU	1.40 (0.69–2.85)	0.348	1.39 (0.67–2.90)	0.378	1.74 (0.79–3.87)	0.172	1.56 (0.66–3.70)	0.316
Moderate-to-vigorous PA, medium AU	0.90 (0.36–2.29)	0.829	0.81 (0.33–2.03)	0.658	0.63 (0.16–2.46)	0.510	0.58 (0.16–2.14)	0.411
Moderate-to-vigorous PA, high AU	1.99 (1.03–3.84)	**0.041**	1.39 (0.70–2.75)	0.343	2.72 (1.08–6.82)	**0.033**	2.00 (0.80-5.00)	0.138
Low PA, occasional AU	1.11 (0.76–1.62)	0.587	1.21 (0.82–1.78)	0.333	1.24 (0.82–1.86)	0.306	1.40 (0.89–2.19)	0.146
Low PA, low AU	0.74 (0.38–1.45)	0.380	0.83 (0.43–1.59)	0.568	0.77 (0.35–1.73)	0.534	0.87 (0.39–1.93)	0.731
Low PA, medium AU	0.97 (0.44–2.14)	0.941	0.90 (0.43–1.86)	0.769	0.61 (0.23–1.56)	0.299	0.57 (0.23–1.45)	0.240
Low PA, high AU	1.64 (1.01–2.68)	**0.047**	1.29 (0.77–2.15)	0.335	2.47 (1.30–4.68)	**0.006**	2.04 (1.11–3.77)	**0.022**
Sedentary PA, occasional AU	2.07 (1.42–3.03)	**< 0.001**	2.18 (1.47–3.23)	**< 0.001**	2.03 (1.33–3.10)	**0.001**	2.22 (1.44–3.42)	**< 0.001**
Sedentary PA, low AU	1.89 (0.92–3.89)	0.082	2.04 (1.00-4.17)	0.051	1.55 (0.55–4.37)	0.405	1.67 (0.61–4.58)	0.319
Sedentary PA, medium AU	2.57 (1.14–5.79)	**0.023**	2.85 (1.26–6.45)	**0.012**	2.26 (0.82–6.23)	0.114	2.72 (1.08–6.85)	**0.034**
Sedentary PA, high AU	2.35 (1.24–4.44)	**0.009**	2.20 (1.16–4.16)	**0.015**	2.90 (1.46–5.75)	**0.002**	2.95 (1.51–5.79)	**0.002**

Abbreviations: PA, physical activity; AU, alcohol use; HR, hazard ratio

^a^ Adjusted for age, race, education, marital status, body mass index, and smoking status

^b^ Adjusted for congestive heart failure, myocardial infarction, stroke, cancer, hypertension, hypercholesterolemia, and diabetes mellitus

^c^ Adjusted for age, race, education, marital status, body mass index, smoking status, congestive heart failure, myocardial infarction, stroke, cancer, hypertension, hypercholesterolemia, and diabetes mellitus

Univariate Cox regression analyses showed that low PA (hazard ratio [HR] = 0.53, 95% confidence interval [CI] 0.42–0.66, *P* < 0.001), and moderate-to-vigorous PA (HR = 0.54, 95% CI 0.41–0.72, *P* < 0.001) acted as protective factors for mortality, while high-volume AU was a risk factor for mortality (HR = 1.45, 95% CI 1.10–1.93, *P* = 0.010. After adjustment for possible confounding factors, the correlation remained significant (all *P* < 0.05).

The integration of high volume AU with any degree of PA, including sedentary PA (HR = 2.35, 95% CI 1.24–4.44, *P* = 0.009), low PA (HR = 1.64, 95% CI 1.01–2.68, *P* = 0.047), and moderate-to-vigorous PA (HR = 1.99, 95% CI 1.03–3.84, *P* = 0.041), was associated with an increased risk of mortality. This relationship persisted as significant after adjusting for potential confounders (all *P* < 0.05). Additionally, occasional AU (HR = 2.07, 95% CI 1.42–3.03, *P* < 0.001) or medium volume AU (HR = 2.57, 95% CI 1.14–5.79, *P* = 0.023), when combined with sedentary lifestyle was also associated with increased mortality risk. After adjustment of possible confounding factors, the association remained significant (all *P* < 0.05).

### The association between PA and AU with all-cause mortality for age ≥ 60 years

For the older participants, the all-cause mortality per 100,000 person-years varied significantly among different PA levels, but no significant difference among different AU levels (P for trend in PA = 0.013, P for trend in AU = 0.246; Fig. 2).

The Cox regression analyses between PA and AU with all-cause mortality for age ≥ 60 years were summarized in Table 3.

**Table 3:** Cox regression analysis of physical activity and alcohol use with all-cause mortality for age ≥ 60 years

Variables	Univariate reanalysis		Adjusted model 1^a^		Adjusted model 2^b^		Adjusted model 3^c^	
	HR (95% CI)	*P* value	HR (95% CI)	*P* value	HR (95% CI)	*P* value	HR (95% CI)	*P* value
**Physical activity**								
Sedentary	Reference		Reference		Reference		Reference	
Low	0.57 (0.51–0.64)	**< 0.001**	0.60 (0.53–0.67)	**< 0.001**	0.64 (0.56–0.74)	**< 0.001**	0.65 (0.57–0.75)	**< 0.001**
Moderate-to-vigorous	0.52 (0.45–0.61)	**< 0.001**	0.52 (0.45–0.62)	**< 0.001**	0.61 (0.51–0.72)	**< 0.001**	0.60 (0.50–0.72)	**< 0.001**
**Alcohol use**								
Occasional	Reference		Reference		Reference		Reference	
Low volume	0.81 (0.68–0.97)	**0.020**	0.80 (0.66–0.96)	**0.018**	0.91 (0.77–1.09)	0.310	0.86 (0.72–1.04)	0.128
Medium volume	1.06 (0.82–1.36)	0.667	1.02 (0.80–1.31)	0.875	1.15 (0.87–1.52)	0.340	1.12 (0.84–1.49)	0.425
High volume	0.98 (0.70–1.36)	0.887	0.85 (0.59–1.21)	0.368	0.95 (0.66–1.36)	0.767	0.85 (0.59–1.23)	0.384
**Combined variables**								
Moderate-to-vigorous PA, occasional AU	Reference		Reference		Reference		Reference	
Moderate-to-vigorous PA, low AU	0.59 (0.37–0.94)	**0.027**	0.58 (0.36–0.94)	**0.025**	0.59 (0.37–0.94)	**0.026**	0.56 (0.35–0.89)	**0.015**
Moderate-to-vigorous PA, medium AU	1.12 (0.65–1.93)	0.676	1.22 (0.74–2.01)	0.431	1.26 (0.73–2.16)	0.405	1.41 (0.83–2.38)	0.204
Moderate-to-vigorous PA, high AU	0.76 (0.43–1.33)	0.336	0.80 (0.41–1.56)	0.513	0.73 (0.37–1.43)	0.357	0.68 (0.31–1.49)	0.338
Low PA, occasional AU	1.03 (0.85–1.26)	0.749	1.10 (0.92–1.32)	0.295	0.98 (0.81–1.19)	0.864	1.03 (0.86–1.25)	0.736
Low PA, low AU	0.90 (0.64–1.25)	0.530	0.92 (0.66–1.28)	0.620	0.96 (0.69–1.32)	0.792	0.94 (0.68–1.29)	0.690
Low PA, medium AU	1.27 (0.85–1.90)	0.249	1.33 (0.89–1.98)	0.168	1.18 (0.77–1.82)	0.446	1.24 (0.81–1.88)	0.316
Low PA, high AU	1.19 (0.75–1.89)	0.469	1.09 (0.68–1.75)	0.707	1.23 (0.77–1.96)	0.397	1.15 (0.72–1.83)	0.566
Sedentary PA, occasional AU	1.83 (1.54–2.18)	**< 0.001**	1.90 (1.59–2.27)	**< 0.001**	1.56 (1.29–1.88)	**< 0.001**	1.62 (1.34–1.97)	**< 0.001**
Sedentary PA, low AU	1.91 (1.38–2.64)	**< 0.001**	1.89 (1.35–2.66)	**< 0.001**	1.91 (1.30–2.82)	**< 0.001**	1.87 (1.27–2.74)	**0.001**
Sedentary PA, medium AU	1.46 (0.95–2.27)	0.088	1.17 (0.74–1.86)	0.509	1.59 (1.06–2.39)	**0.025**	1.31 (0.79–2.16)	0.300
Sedentary PA, high AU	1.56 (0.89–2.76)	0.124	1.14 (0.63–2.09)	0.660	0.74 (0.29–1.84)	0.512	0.65 (0.27–1.52)	0.318

Abbreviations: PA, physical activity; AU, alcohol use; HR, hazard ratio

^a^ Adjusted for age, race, education, marital status, body mass index, and smoking status

^b^ Adjusted for congestive heart failure, myocardial infarction, stroke, cancer, hypertension, hypercholesterolemia, and diabetes mellitus

^c^ Adjusted for age, race, education, marital status, body mass index, smoking status, congestive heart failure, myocardial infarction, stroke, cancer, hypertension, hypercholesterolemia, and diabetes mellitus

Similar to young participants, maintaining physically active lifestyle, including low PA (HR = 0.57, 95% CI 0.51–0.64, *P* < 0.001), and moderate-to-vigorous PA (HR = 0.52, 95% CI 0.45–0.61, *P* < 0.001), was correlated with lower death risk in old population. After adjustment, the beneficial effect for prognosis remained significant (*P* < 0.05). In regards to alcohol consumption, low volume AU were related to lower risk of death (HR = 0.81, 95% CI 0.68–0.97, *P* = 0.020). The significance of this relationship dissipated after adjusting for confounders, including demographic variables and medical conditions (*P* > 0.05).

In addition, the combination of a sedentary lifestyle with either occasional AU (HR = 1.83, 95% CI 1.54–2.18, *P* < 0.001), or low AU (HR = 1.91, 95% CI 1.38–2.64, *P* < 0.001) was associated with an increased risk of mortality in old people, which remained significant after adjustment (*P* < 0.05). Finally, it is noteworthy that, combining moderate-to-vigorous PA and low volume AU (HR = 0.59, 95% CI 0.37–0.94, *P* = 0.027) in old participants was associated with a reduction in mortality. After adjustment, the combination of moderate-to-vigorous PA and low volume AU was independently associated with favorable prognostic outcomes (all *P* < 0.05).

## Discussion

Physically active individuals report higher frequency and quantity of alcohol consumption [12]. Dodge et al. conducted a systemic review on the relationship between PA and AU among adults in the United States and concluded that they were positively correlated [22]. Studying the combined effects of PA and AU is crucial for understanding their influence on long-term mortality and providing evidence for public health promotion that targets PA and AU, but research in this field is scarce. To the best of our knowledge, this study is the first to explore the combined association of PA and AU with long-term mortality. The major findings of our study revealed that, across both young and old populations, being physically active consistently contributed to positive health outcomes. A sedentary lifestyle combined with even low levels of AU was associated with higher mortality risk. Specifically, among young adults, the combination of any level of PA with high volume AU was linked to unfavorable outcomes. For old participants, engaging in moderate-to-vigorous PA while low volume AU emerged as a beneficial practice.

### The effect of PA on mortality

The current PA guidelines for young and old populations are similar. They recommend at least 150–300 min per week of moderate PA, or at least 75–150 min per week of vigorous PA, or an equivalent combination of both [5–7, 23]. An inverse curvilinear dose–response relationship is observed between weekly PA level and the reduction of mortality risk [24]. A prospective cohort study conducted by Lee et al. demonstrated a 19% risk reduction for all-cause mortality for participants with vigorous PA, and a 20% risk reduction for those with moderate PA, over a median follow-up of 26 years [25]. Consistent with previous studies, our study found that PA, even at low level, correlated with decreased long-term all-cause mortality, with adjustments for confounders.

### The effect of AU on mortality

Recommendations for alcohol consumption have not reached a consensus opinion. Evidence from observational and prospective studies have consistently shown a lower risk of mortality in people with low-volume AU when compared with abstainers or individuals with high volume AU, in a J-shaped curve relationship [26–29]. First, compared with high volume AU, low-to-medium volume AU may prevent the development of heart failure [30, 31], coronary artery disease, myocardial infarction, peripheral arterial disease, and stroke [32]. Second, high volume AU is associated with chronic liver disease [33], atrial fibrillation [34], dilated cardiomyopathy [35], and cancer [36, 37]. However, the threshold at which the volume of AU shifts from being beneficial to detrimental for health is not clearly defined [38]. Additionally, many confounding factors could affect the relationship between AU and outcomes. A meta-analysis showed a non-significant reduction in mortality risk in people with low-volume AU, probably because of the abstainer bias, that is to say, the abstainers with whom they were compared quitted drinking because of pre-existing medical conditions caused by prior high volume AU [39, 40]. In our study, low volume AU was associated with survival benefit; however, after adjustment, the beneficial effect disappeared.

### The combined association of PA and AU with mortality

Our results demonstrated a lower mortality risk for old participants who had moderate-to-vigorous PA and low volume AU, and a higher mortality risk for young participants who had moderate-to-vigorous PA and high volume AU. The underlying mechanism is yet unclear, but several possible explanations exist.

Firstly, exercise benefits clinical outcomes irrespective of traditional risk factors. Regular exercise facilitates cardiac parasympathetic regulation, generates a healthy anti-inflammatory environment, increases circulating angiogenic cells, and protects the gut barrier [41]. By contrast, sarcopenia, defined as the loss of muscle strength, mass, and function, is related to a higher risk of mortality, falls, and compromised quality of life [42]. Secondly, low volume AU can contribute to an increase in high-density lipoprotein, fibrinolysis, and endothelial function, and a decrease in platelet aggregation and plasma viscosity [26, 43]. These factors might account for the net decrease in mortality risk observed in old participants when moderate-to-vigorous PA and low volume AU were combined.

Nevertheless, we saw an increased risk of mortality in young participants when moderate-to-vigorous PA was combined with high volume AU. The equilibrium of skeletal muscle protein synthesis and degradation is partially regulated by the rapamycin complex 1 (mTORC1) signaling pathway [44, 45]. Alcohol and exercise could independently influence this pathway, but in opposite directions; that is, alcohol generally has a suppressive effect on this pathway and causes muscle losses, while exercise has a stimulatory effect and leads to muscle gains [15, 46–48]. Therefore, the harmful effect of chronic high volume AU might initially antagonize and gradually override the beneficial effect associated with regular exercise, such as increases in mTORC1 pathway signaling, gains in muscle size and decrease in long term mortality [14].

Our study had several limitations. First, this study was an observational cohort study. Though multivariate hazard regression models were used, residual confounding factors may exist. Randomized controlled trials are required to testify our results. Second, the evaluation of PA and AU was not comprehensive because of limited variables. Future studies could involve information on the intensity and type of PA, or the timing and frequency of AU. Third, while our study revealed the combined effect of PA and AU on mortality, the underlying biological process driving the association are as yet unknown. Further experimental studies are necessary to elucidate the mechanisms.

## Conclusions

As compared to sedentary lifestyle, being physically active decreases mortality in both young and old populations. Combining sedentary lifestyle with even minimal alcohol consumption correlated with elevated risk of death. In young group, combining any level of PA with high volume AU was associated with increased mortality. In old group, combining moderate-to-vigorous PA with low volume AU was related to reduced mortality. These findings demonstrated the possible relationships between different levels of PA and AU and long-term mortality, which may provide guidance for public health promotion strategies that target PA and AU.

### Electronic supplementary material

Below is the link to the electronic supplementary material.

Supplementary Material 1

## Data Availability

The NHANES datasets are available online at: https://wwwn.cdc.gov/nchs/nhanes/Default.aspx. Death data were extracted from public-use linked mortality files in the NHANSE database, which is available at: https://www.cdc.gov/nchs/data-linkage/mortality-public.htm.

## Abbreviations

PA: Physical activity
AU: Alcohol use
BMI: Body mass index
mTORC1: Rapamycin complex 1
HR: Hazard ratio
CI: Confidential interval
