# Supplementary material for: The combined association of physical activity and alcohol use with long-term mortality: an age-stratified analysis
Source: BMC Public Health. 2024 Jul 8;24:1817. doi: 10.1186/s12889-024-19326-8 (PMC11229205; doi:10.1186/s12889-024-19326-8)
Supplement: Supplementary file 1 — Supplementary Material 1 [file 12889_2024_19326_MOESM1_ESM.docx]

**Supplementary table 1. Baseline characteristics for age < 60 years, based on levels of PA**

| **Characteristics** | **Sedentary**  (N = 1854) | **Low**  (N = 4237) | **Moderate-to-vigorous**  (N = 2167) | **P value** |
| --- | --- | --- | --- | --- |
|  | **Unweighted N (weighted percentage)** | | |  |
| **Male gender** | 754 (44.3) | 1751 (44.5) | 1323 (60.9) | <0.001 |
| **Race** |  |  |  | <0.001 |
| Non-Hispanic White | 947 (70.5) | 1796 (66.3) | 1132 (74.0) |  |
| Non-Hispanic Black | 449 (13.7) | 882 (12.8) | 368 (9.3) |  |
| Mexican American | 284 (5.7) | 1140 (9.9) | 506 (8.7) |  |
| Other Hispanic | 95 (5.2) | 231 (6.2) | 94 (4.3) |  |
| Others | 79 (4.9) | 188 (5.6) | 67 (3.7) |  |
| **BMI group** |  |  |  | <0.001 |
| Underweight | 37 (2.3) | 67 (1.7) | 42 (2.6) |  |
| Normal | 526 (30.1) | 1345 (34.4) | 752 (36.0) |  |
| Overweight | 589 (31.3) | 1415 (31.4) | 781 (35.5) |  |
| Obese | 702 (36.3) | 1410 (32.5) | 592 (25.9) |  |
| **Education** |  |  |  | <0.001 |
| < high school | 440 (17.3) | 1203 (18.0) | 572 (17.2) |  |
| high school | 396 (21.7) | 965 (23.4) | 616 (30.6) |  |
| > high school | 1018 (61.1) | 2069 (58.6) | 979 (52.2) |  |
| **Marital status** |  |  |  | 0.014 |
| Married | 979 (55.8) | 2387 (57.4) | 1218 (53.8) |  |
| Widowed | 28 (1.1) | 70 (1.4) | 23 (1.2) |  |
| Divorced | 196 (11.4) | 366 (9.2) | 151 (8.5) |  |
| Separated | 80 (3.3) | 172 (3.6) | 80 (2.8) |  |
| Never married | 422 (21.2) | 945 (22.3) | 493 (23.9) |  |
| Living with partner | 149 (7.1) | 297 (6.1) | 202 (9.7) |  |
| **Smoking status** |  |  |  | 0.073 |
| Never smoker | 1003 (53.1) | 2377 (52.0) | 1077 (48.1) |  |
| Former smoker | 375 (21.2) | 767 (19.8) | 438 (21.6) |  |
| Current smoker | 476 (25.7) | 1093 (28.2) | 652 (30.4) |  |
| **Alcohol use** |  |  |  | 0.002 |
| Occasional | 1460 (77.7) | 3282 (76.5) | 1558 (70.7) |  |
| Low volume | 154 (8.9) | 354 (8.7) | 199 (10.7) |  |
| Medium volume | 90 (5.8) | 250 (6.6) | 159 (7.1) |  |
| High volume | 150 (7.6) | 351 (8.2) | 251 (11.4) |  |
| **Past medical history** |  |  |  |  |
| Congestive heart failure | 34 (1.8) | 37 (1.0) | 10 (0.6) | 0.032 |
| Coronary heart disease | 37 (2.3) | 53 (1.5) | 19 (1.1) | 0.133 |
| Angina | 42 (2.5) | 43 (1.3) | 27 (1.5) | 0.042 |
| Myocardial infarction | 42 (2.6) | 53 (1.7) | 25 (1.3) | 0.076 |
| Stroke | 37 (1.9) | 44 (1.2) | 9 (0.8) | 0.048 |
| Cancer | 88 (6.1) | 153 (4.7) | 61 (3.5) | 0.036 |
| Hypertension | 459 (24.9) | 791 (20.0) | 356 (17.8) | <0.001 |
| Hypercholesterolemia | 423 (35.6) | 774 (32.1) | 338 (29.3) | 0.043 |
| Diabetes mellitus | 129 (6.4) | 212 (4.8) | 74 (2.6) | 0.001 |
| Chronic kidney disease | 34 (2.3) | 41 (0.9) | 18 (1.3) | 0.021 |
|  | **Weighted mean (SD)** | | |  |
| Age (years) | 40.36 (10.79) | 39.09 (11.22) | 36.85 (11.06) | <0.001 |
| BMI (kg/m^2^) | 29.11 (7.58) | 28.08 (6.48) | 27.28 (5.74) | <0.001 |
| SBP (mmHg) | 119.45 (15.74) | 119.80 (16.43) | 119.10 (14.90) | 0.512 |
| DBP (mmHg) | 73.37 (11.20) | 72.86 (12.15) | 72.16 (12.26) | 0.176 |
| WBC count (/mm^3^) | 7.38 (2.22) | 7.41 (2.11) | 7.34 (2.15) | 0.545 |
| Hemoglobin (g/dL) | 14.42 (1.47) | 14.46 (1.49) | 14.87 (1.48) | <0.001 |
| Lymphocyte count (/mm^3^) | 2.17 (0.82) | 2.18 (0.68) | 2.16 (0.73) | 0.798 |
| Platelet count (/mm^3^) | 275.19 (70.01) | 272.10 (64.91) | 268.98 (60.44) | 0.057 |
| TC (mmol/L) | 5.20 (1.13) | 5.18 (1.08) | 5.17 (1.17) | 0.919 |
| LDL-C (mmol/L) | 3.05 (0.85) | 3.10 (0.89) | 3.10 (0.99) | 0.474 |
| HDL-C (mmol/L) | 1.38 (0.41) | 1.39 (0.42) | 1.38 (0.40) | 0.646 |
| Total bilirubin (umol/L) | 11.80 (4.97) | 12.01 (5.04) | 12.91 (5.60) | <0.001 |
| Albumin (g/L) | 43.29 (3.43) | 43.54 (3.50) | 44.34 (3.44) | <0.001 |
| Creatinine (umol/L) | 74.66 (38.43) | 71.65 (25.78) | 75.58 (35.72) | 0.001 |
| Glycohemoglobin (%) | 5.42 (0.91) | 5.40 (0.87) | 5.30 (0.69) | <0.001 |

Abbreviations: NHANES, National Health and Nutrition Examination Survey; BMI, body mass index; SBP, systolic blood pressure; DBP, diastolic blood pressure; SD, standard deviation; PA, physical activity; AU, alcohol use; WBC, white blood cell; TC, total cholesterol; LDL-C, low-density lipoprotein cholesterol; HDL-C, high-density lipoprotein cholesterol.

**Supplementary table 2. Baseline characteristics for age ≥ 60 years, based on levels of PA**

| **Characteristics** | **Sedentary**  (N = 1277) | | **Low**  (N = 2490) | **Moderate-to-vigorous**  (N = 596) | **P value** |  |
| --- | --- | --- | --- | --- | --- | --- |
|  | | **Unweighted N (weighted percentage)** | | |  | |
| **Male gender** | 629 (44.6) | | 1200 (42.3) | 342 (53.8) | 0.001 |  |
| **Race** |  | |  |  | <0.001 |  |
| Non-Hispanic White | 688 (76.9) | | 1414 (81.7) | 405 (88.6) |  |  |
| Non-Hispanic Black | 282 (12.3) | | 339 (6.7) | 71 (4.9) |  |  |
| Mexican American | 227 (2.9) | | 584 (3.8) | 96 (2.1) |  |  |
| Other Hispanic | 45 (4.3) | | 88 (4.2) | 15 (2.7) |  |  |
| Others | 35 (3.6) | | 65 (3.6) | 9 (1.7) |  |  |
| **BMI group** |  | |  |  | <0.001 |  |
| Underweight | 21 (1.8) | | 26 (1.0) | 7 (1.4) |  |  |
| Normal | 312 (23.5) | | 716 (29.9) | 173 (26.9) |  |  |
| Overweight | 467 (34.2) | | 1020 (39.9) | 259 (44.8) |  |  |
| Obese | 477 (40.4) | | 728 (29.2) | 157 (26.8) |  |  |
| **Education** |  | |  |  | <0.001 |  |
| < high school | 619 (39.5) | | 1032 (27.1) | 184 (19.6) |  |  |
| high school | 281 (25.8) | | 590 (29.9) | 143 (26.6) |  |  |
| > high school | 377 (34.7) | | 868 (43.1) | 269 (53.8) |  |  |
| **Marital status** |  | |  |  | <0.001 |  |
| Married | 635 (53.4) | | 1544 (64.4) | 406 (70.2) |  |  |
| Widowed | 414 (30.2) | | 583 (21.8) | 100 (15.6) |  |  |
| Divorced | 129 (10.3) | | 211 (9.0) | 58 (9.7) |  |  |
| Separated | 29 (1.5) | | 48 (1.1) | 13 (1.1) |  |  |
| Never married | 54 (3.6) | | 73 (2.6) | 13 (2.2) |  |  |
| Living with partner | 16 (0.9) | | 31 (1.1) | 6 (1.2) |  |  |
| **Smoking status** |  | |  |  | 0.028 |  |
| Never smoker | 564 (43.5) | | 1203 (48.1) | 265 (43.7) |  |  |
| Former smoker | 536 (41.6) | | 1021 (41.8) | 246 (44.4) |  |  |
| Current smoker | 177 (14.9) | | 265 (10.2) | 85 (12.0) |  |  |
| **Alcohol use** |  | |  |  | 0.070 |  |
| Occasional | 1110 (86.4) | | 2070 (81.3) | 474 (79.8) |  |  |
| Low volume | 88 (6.7) | | 234 (10.0) | 64 (10.7) |  |  |
| Medium volume | 40 (3.5) | | 94 (4.3) | 33 (5.5) |  |  |
| High volume | 39 (3.4) | | 92 (4.4) | 25 (4.0) |  |  |
| **Past medical history** |  | |  |  |  |  |
| Congestive heart failure | 150 (13.2) | | 136 (4.9) | 23 (2.8) | <0.001 |  |
| Coronary heart disease | 161 (13.1) | | 235 (10.8) | 57 (10.3) | 0.249 |  |
| Angina | 132 (10.9) | | 194 (9.3) | 37 (6.1) | 0.028 |  |
| Myocardial infarction | 178 (13.5) | | 225 (9.9) | 45 (8.3) | 0.025 |  |
| Stroke | 135 (10.0) | | 149 (5.5) | 32 (5.0) | 0.002 |  |
| Cancer | 250 (22.3) | | 439 (21.0) | 111 (22.7) | 0.682 |  |
| Hypertension | 785 (61.5) | | 1294 (50.5) | 279 (46.3) | <0.001 |  |
| Hypercholesterolemia | 509 (50.5) | | 1100 (53.1) | 260 (53.6) | O.514 |  |
| Diabetes mellitus | 340 (24.7) | | 395 (13.2) | 75 (9.7) | <0.001 |  |
| Chronic kidney disease | 61 (6.4) | | 61 (3.1) | 8 (2.3) | 0.002 |  |
|  | **Weighted mean (SD)** | | | |  |  |
| Age (years) | 71.80 (8.03) | | 70.12 (7.19) | 69.11 (6.84) | <0.001 |  |
| BMI (kg/m^2^) | 29.41 (6.36) | | 27.82 (5.11) | 27.82 (4.86) | <0.001 |  |
| SBP (mmHg) | 139.15 (22.93) | | 141.55 (22.98) | 139.18 (21.85) | 0.054 |  |
| DBP (mmHg) | 68.08 (16.43) | | 70.06 (15.84) | 70.92 (16.82) | 0.063 |  |
| WBC count (/mm^3^) | 7.46 (2.10) | | 7.05 (2.61) | 7.10 (3.62) | 0.001 |  |
| Hemoglobin (g/dL) | 14.09 (1.47) | | 14.28 (1.31) | 14.43 (1.30) | <0.001 |  |
| Lymphocyte count (/mm^3^) | 2.00 (0.84) | | 2.05 (1.94) | 2.13 (3.06) | 0.512 |  |
| Platelet count (/mm^3^) | 256.19 (72.13) | | 256.16 (68.25） | 255.96 (76.66) | 0.999 |  |
| TC (mmol/L) | 5.38 (1.11) | | 5.45 (1.08) | 5.51 (1.04) | 0.245 |  |
| LDL-C (mmol/L) | 3.13 (0.88) | | 3.23 (0.99) | 3.25 (0.88) | 0.203 |  |
| HDL-C (mmol/L) | 1.39 (0.44) | | 1.45 (0.42) | 1.43 (0.43) | 0.099 |  |
| Total bilirubin (umol/L) | 11.86 (5.37) | | 12.26 (6.24) | 12.45 (4.88) | 0.187 |  |
| Albumin (g/L) | 41.61 (3.27) | | 42.54 (2.92) | 42.83 (2.87) | <0.001 |  |
| Creatinine (umol/L) | 90.04 (60.68) | | 81.96 (39.64) | 81.08 (28.68) | <0.001 |  |
| Glycohemoglobin (%) | 5.96 (1.13) | | 5.76 (0.98) | 5.75 (0.92) | <0.001 |  |

Abbreviations: NHANES, National Health and Nutrition Examination Survey; BMI, body mass index; SBP, systolic blood pressure; DBP, diastolic blood pressure; SD, standard deviation; PA, physical activity; AU, alcohol use; WBC, white blood cell; TC, total cholesterol; LDL-C, low-density lipoprotein cholesterol; HDL-C, high-density lipoprotein cholesterol.

**Supplementary table 3. Baseline characteristics for age < 60 years, based on levels of AU**

| **Characteristics** | **Occasional**  (N = 6300) | **Low volume**  (N = 707) | **Medium volume**  (N = 499) | **High volume**  (N = 752) | **P value** |
| --- | --- | --- | --- | --- | --- |
|  | **Unweighted N (weighted percentage)** | | | |  |
| **Male gender** | 2540 (43.8) | 371 (51.0) | 344 (69.8) | 573 (75.3) | <0.001 |
| **Race** |  |  |  |  | 0.004 |
| Non-Hispanic White | 2849 (67.5) | 379 (75.7) | 256 (74.6) | 391 (75) |  |
| Non-Hispanic Black | 1306 (12.2) | 120 (8.8) | 118 (11.7) | 155 (9.8) |  |
| Mexican American | 1529 (9.0) | 158 (7.1) | 89 (6.6) | 154 (7.6) |  |
| Other Hispanic | 340 (5.8) | 28 (4.5) | 23 (4.9) | 29 (3.8) |  |
| Others | 276 (5.4) | 22 (3.9) | 13 (2.2) | 23 (3.9) |  |
| **BMI group** |  |  |  |  | <0.001 |
| Underweight | 109 (2.1) | 16 (2.4) | 7 (1.0) | 14 (2.8) |  |
| Normal | 1889 (31.8) | 260 (40.0) | 199 (44.2) | 275 (37.0) |  |
| Overweight | 2051 (31.2) | 259 (33.7) | 182 (36.8) | 293 (38.8) |  |
| Obese | 2251 (34.9) | 172 (23.9) | 111 (18.0) | 170 (21.4) |  |
| **Education** |  |  |  |  | <0.001 |
| < high school | 1734 (18.5) | 163 (13.3) | 101 (11.9) | 217 (18.3) |  |
| high school | 1530 (25.5) | 129 (18.7) | 128 (25.0) | 190 (27.6) |  |
| > high school | 3036 (56.0) | 415 (68.0) | 270 (63.1) | 345 (54.1) |  |
| **Marital status** |  |  |  |  | 0.002 |
| Married | 3582 (57.2) | 410 (59.8) | 261 (52.2) | 331 (45.0) |  |
| Widowed | 100 (1.4) | 7 (0.8) | 7 (1.8) | 7 (0.8) |  |
| Divorced | 515 (9.0) | 69 (10.2) | 45 (9.5) | 84 (13.3) |  |
| Separated | 259 (3.4) | 22 (2.6) | 18 (3.0) | 33 (4.1) |  |
| Never married | 1397 (22.4) | 136 (18.7) | 117 (22.3) | 210 (27.2) |  |
| Living with partner | 447 (6.7) | 63 (7.9) | 51 (11.2) | 87 (9.7) |  |
| **Smoking status** |  |  |  |  | <0.001 |
| Never smoker | 3673 (54.7) | 339 (48.1) | 198 (41.6) | 247 (31.9) |  |
| Former smoker | 1178 (19.9) | 149 (23.7) | 119 (26.7) | 134 (19.0) |  |
| Current smoker | 1449 (25.4) | 219 (28.3) | 182 (31.7) | 371 (49.1) |  |
| **Physical activity** |  |  |  |  | 0.002 |
| Sedentary | 1460 (24.4) | 154 (22.5) | 90 (21.0) | 150 (20.0) |  |
| Low | 3282 (49.6) | 354 (45.6) | 250 (48.9) | 351 (44.7) |  |
| Moderate-to-vigorous | 1558 (26.0) | 199 (31.9) | 159 (30.1) | 251 (35.3) |  |
| **Past medical history** |  |  |  |  |  |
| Congestive heart failure | 68 (1.2) | 3 (0.4) | 4 (0.5) | 6 (1.0) | 0.257 |
| Coronary heart disease | 85 (1.6) | 4 (0.6) | 10 (2.2) | 10 (1.8) | 0.285 |
| Angina | 93 (1.8) | 3 (0.7) | 6 (1.0) | 10 (1.7) | 0.294 |
| Myocardial infarction | 94 (1.9) | 5 (0.9) | 7 (1.4) | 14 (2.3) | 0.365 |
| Stroke | 76 (1.4) | 5 (0.9) | 1 (0.0) | 8 (0.9) | 0.041 |
| Cancer | 237 (4.7) | 27 (4.7) | 18 (5.4) | 20 (3.9) | 0.790 |
| Hypertension | 1258 (21.2) | 111 (16.4) | 96 (21.2) | 141 (19.5) | 0.21 |
| Hypercholesterolemia | 1181 (32.7) | 123 (26.3) | 101 (35.0) | 130 (34.2) | 0.128 |
| Diabetes mellitus | 361 (5.2) | 26 (3.6) | 9 (1.9) | 19 (2.0) | 0.012 |
| Chronic kidney disease | 79 (1.5) | 8 (1.1) | 2 (0.5) | 4 (1.0) | 0.579 |
|  | **Weighted mean (SD)** | | | |  |
| Age (years) | 38.58 (11.32) | 39.80 (10.51) | 39.95 (10.57) | 38.44 (10.67) | 0.074 |
| BMI (kg/m^2^) | 28.58 (6.87) | 26.76 (5.87) | 26.32 (5.07) | 26.82 (5.32) | <0.001 |
| SBP (mmHg) | 119.05 (16.04) | 119.31 (15.73) | 121.04 (14.69) | 122.49 (14.92) | 0.001 |
| DBP (mmHg) | 72.60 (11.99) | 72.41 (11.32) | 74.47 (11.86) | 73.38 (12.48) | 0.119 |
| WBC count (/mm^3^) | 7.48 (2.20) | 7.04 (1.86) | 6.98 (1.97) | 7.22 (2.09) | <0.001 |
| Hemoglobin (g/dL) | 14.45 (1.51) | 14.56 (1.43) | 14.92 (1.34) | 15.20 (1.32) | <0.001 |
| Lymphocyte count (/mm^3^) | 2.20 (0.74) | 2.11 (0.69) | 2.10 (0.67) | 2.06 (0.66) | <0.001 |
| Platelet count (/mm^3^) | 274.52 (66.28) | 265.86 (61.53) | 257.92 (57.82) | 267.16 (60.44) | <0.001 |
| TC (mmol/L) | 5.16 (1.14) | 5.17 (0.93) | 5.26 (1.02) | 5.31 (1.16) | 0.050 |
| LDL-C (mmol/L) | 3.10 (0.91) | 3.01 (0.90) | 3.04 (0.86) | 3.14 (0.94) | 0.523 |
| HDL-C (mmol/L) | 1.35 (0.40) | 1.48 (0.43) | 1.50 (0.47) | 1.47 (0.41) | 0.003 |
| Total bilirubin (umol/L) | 11.98 (5.24) | 12.69 (5.35) | 13.26 (4.84) | 12.93 (4.86) | <0.001 |
| Albumin (g/L) | 43.47 (3.52) | 44.16 (3.41) | 44.40 (3.11) | 44.71 (3.33) | <0.001 |
| Creatinine (umol/L) | 72.62 (35.69) | 73.41 (17.24) | 78.38 (16.46) | 76.76 (15.62) | <0.001 |
| Glycohemoglobin (%) | 5.41 (0.88) | 5.30 (0.63) | 5.25 (0.61) | 5.26 (0.74) | <0.001 |

Abbreviations: NHANES, National Health and Nutrition Examination Survey; BMI, body mass index; SBP, systolic blood pressure; DBP, diastolic blood pressure; SD, standard deviation; PA, physical activity; AU, alcohol use; WBC, white blood cell; TC, total cholesterol; LDL-C, low-density lipoprotein cholesterol; HDL-C, high-density lipoprotein cholesterol.

**Supplementary table 4. Baseline characteristics for age ≥ 60 years, based on levels of AU**

| **Characteristics** | **Occasional**  (N = 3654) | **Low volume**  (N = 386) | **Medium volume**  (N = 167) | **High volume**  (N = 156) | **P value** |
| --- | --- | --- | --- | --- | --- |
|  | **Unweighted N (weighted percentage)** | | | |  |
| **Male gender** | 1689 (41.1) | 231 (54.8) | 122 (63.5) | 129 (76.6) | <0.001 |
| **Race** |  |  |  |  | 0.030 |
| Non-Hispanic White | 2029 (80.2) | 260 (85.9) | 113 (88.5) | 105 (88.9) |  |
| Non-Hispanic Black | 598 (8.5) | 44 (4.8) | 24 (5.3) | 26 (7.5) |  |
| Mexican American | 804 (3.5) | 61 (2.2) | 22 (2.2) | 20 (1.6) |  |
| Other Hispanic | 128 (4.2) | 12 (4.5) | 5 (2.5) | 3 (1.2) |  |
| Others | 95 (3.6) | 9 (2.6) | 3 (1.5) | 2 (0.9) |  |
| **BMI group** |  |  |  |  | 0.031 |
| Underweight | 43 (1.3) | 6 (1.3) | 2 (0.3) | 3 (2.0) |  |
| Normal | 981 (26.9) | 128 (34.0) | 48 (33.5) | 44 (23.8) |  |
| Overweight | 1453 (38.5) | 158 (42.2) | 67 (37.3) | 68 (46.4) |  |
| Obese | 1177 (33.3) | 94 (22.5) | 50 (28.8) | 41 (27.7) |  |
| **Education** |  |  |  |  | <0.001 |
| < high school | 1630 (32.0) | 117 (17.9) | 50 (14.9) | 38 (15.6) |  |
| high school | 858 (29.1) | 88 (27.4) | 29 (17.5) | 39 (24.0) |  |
| > high school | 1166 (38.9) | 181 (54.6) | 88 (67.6) | 79 (60.3) |  |
| **Marital status** |  |  |  |  | <0.001 |
| Married | 2142 (60.8) | 238 (69.6) | 114 (74.2) | 91 (64.0) |  |
| Widowed | 956 (24.9) | 91 (18.0) | 30 (13.5) | 20 (10.4) |  |
| Divorced | 320 (9.1) | 33 (8.9) | 18 (9.8) | 27 (17.4) |  |
| Separated | 78 (1.3) | 7 (0.8) | 1 (0.1) | 4 (1.5) |  |
| Never married | 119 (2.9) | 11 (2.2) | 1 (0.2) | 9 (4.7) |  |
| Living with partner | 39 (1.0) | 6 (0.6) | 3 (2.1) | 5 (1.9) |  |
| **Smoking status** |  |  |  |  | <0.001 |
| Never smoker | 1815 (49.4) | 150 (40.6) | 42 (23.3) | 25 (15.5) |  |
| Former smoker | 1455 (40.2) | 180 (48.3) | 84 (51.5) | 84 (57.6) |  |
| Current smoker | 383 (10.4) | 56 (11.2) | 41 (25.2) | 47 (26.9) |  |
| **Physical activity** |  |  |  |  | 0.070 |
| Sedentary | 1110 (28.5) | 88 (19.7) | 40 (22.1) | 39 (23.0) |  |
| Low | 2070 (56.5) | 234 (62.3) | 94 (57.9) | 92 (61.6) |  |
| Moderate-to-vigorous | 474 (15.0) | 64 (17.9) | 33 (20.0) | 25 (15.4) |  |
| **Past medical history** |  |  |  |  |  |
| Congestive heart failure | 282 (7.6) | 12 (1.9) | 9 (4.5) | 6 (4.4) | 0.003 |
| Coronary heart disease | 387 (11.8) | 36 (9.5) | 16 (8.3) | 14 (9.5) | 0.562 |
| Angina | 317 (9.8) | 28 (6.9) | 11 (8.0) | 7 (4.4) | 0.163 |
| Myocardial infarction | 377 (10.7) | 36 (9.5) | 19 (11.8) | 16 (11.5) | 0.922 |
| Stroke | 283 (7.5) | 21 (3.5) | 7 (2.3) | 5 (1.4) | <0.001 |
| Cancer | 660 (21.4) | 72 (20.4) | 39 (25.8) | 29 (23.0) | 0.638 |
| Hypertension | 2007 (53.7) | 197 (50.6) | 82 (47.6) | 72 (44.0) | 0.161 |
| Hypercholesterolemia | 1584 (53.0) | 172 (52.7) | 59 (49.0) | 54 (46.3) | 0.587 |
| Diabetes mellitus | 738 (17.5) | 43 (8.3) | 14 (6.8) | 15 (6.9) | <0.001 |
| Chronic kidney disease | 116 (4.1) | 7 (2.9) | 1 (0.5) | 6 (4.8) | 0.295 |
|  | **Weighted mean (SD)** | | | |  |
| Age (years) | 70.63 (7.49) | 70.34 (7.19) | 68.99 (7.33) | 67.90 (6.22) | <0.001 |
| BMI (kg/m^2^) | 28.44 (5.64) | 27.07 (4.63) | 27.42 (4.79) | 27.95 (4.45) | 0.001 |
| SBP (mmHg) | 140.47 (22.92) | 141.75 (23.28) | 138.79 (21.42) | 141.22 (21.04) | 0.567 |
| DBP (mmHg) | 69.17 (16.50) | 71.14 (13.86) | 74.62 (12.27) | 71.39 (17.28) | 0.001 |
| WBC count (/mm^3^) | 7.23 (2.82) | 6.76 (1.79) | 6.96 (1.83) | 7.06 (1.94) | 0.004 |
| Hemoglobin (g/dL) | 14.17 (1.34) | 14.46 (1.30) | 14.77 (1.38) | 14.91 (1.41) | <0.001 |
| Lymphocyte count (/mm^3^) | 2.08 (2.13) | 1.86 (0.68) | 1.91 (0.82) | 1.93 (0.69) | 0.004 |
| Platelet count (/mm^3^) | 258.28 ( 71.96) | 245.17 (62.17) | 243.48 (66.50) | 251.24 (63.15) | 0.001 |
| TC (mmol/L) | 5.45 (1.11) | 5.44 (1.02) | 5.27 (0.92) | 5.55 (0.88) | 0.119 |
| LDL-C (mmol/L) | 3.23 (0.98) | 3.20 (0.78) | 2.95 (0.77) | 3.13 (0.76) | 0.064 |
| HDL-C (mmol/L) | 1.42 (0.42) | 1.45 (0.39) | 1.56 (0.60) | 1.55 (0.43) | 0.027 |
| Total bilirubin (umol/L) | 11.89 (5.83) | 13.01 (4.98) | 14.28 (7.01) | 13.87 (5.11) | <0.001 |
| Albumin (g/L) | 42.19 (3.03) | 42.91 (2.98) | 42.80 (3.04) | 43.47 (3.04) | <0.001 |
| Creatinine (umol/L) | 84.49 (48.31) | 80.66 (23.30) | 80.03 (22.57) | 85.34 (29.40) | 0.055 |
| Glycohemoglobin (%) | 5.88 (1.07) | 5.55 (0.64) | 5.40 (0.53) | 5.53 (0.78) | <0.001 |

Abbreviations: NHANES, National Health and Nutrition Examination Survey; BMI, body mass index; SBP, systolic blood pressure; DBP, diastolic blood pressure; SD, standard deviation; PA, physical activity; AU, alcohol use; WBC, white blood cell; TC, total cholesterol; LDL-C, low-density lipoprotein cholesterol; HDL-C, high-density lipoprotein cholesterol.
